# Metabolic Alterations in Shrimp Stomach During Acute Hepatopancreatic Necrosis Disease and Effects of Taurocholate on *Vibrio parahaemolyticus*

**DOI:** 10.3389/fmicb.2021.631468

**Published:** 2021-04-20

**Authors:** Ramya Kumar, Teng-Chun Tung, Tze Hann Ng, Che-Chih Chang, Yi-Lun Chen, Yi-Min Chen, Shih-Shun Lin, Han-Ching Wang

**Affiliations:** ^1^Department of Biotechnology and Bioindustry Sciences, National Cheng Kung University, Tainan, Taiwan; ^2^International Center for Scientific Development of Shrimp Aquaculture, National Cheng Kung University, Tainan, Taiwan; ^3^Institute for Evolution and Biodiversity, University of Münster, Münster, Germany; ^4^Institute of Biotechnology, National Taiwan University, Taipei, Taiwan

**Keywords:** metabolomics, taurocholate, lipid metabolism, AHPND, *Vibrio parahaemolyticus*, shrimp

## Abstract

Acute hepatopancreatic necrosis disease (AHPND), a recently emerged bacterial shrimp disease, has increased shrimp mortality and caused huge economic losses in many Asian countries. However, molecular factors underlying pathogenesis of this disease remain largely unknown. Our objective was to characterize metabolic alterations in shrimp stomach during AHPND and determine effects of taurocholate on AHPND-causing *Vibrio parahaemolyticus*. Based on metabolomics, pathways for lipid metabolism and for primary bile acid (BA) synthesis were majorly affected following AHPND infection. Bile acid metabolites, namely taurocholate, were downregulated in the metabolomics database. This prompted us to study effects of taurocholate on biofilm formation, PirAB*^vp^* toxin release and biofilm detachment capabilities in AHPND-causing *V. parahaemolyticus*. Treatment of this bacterium with high concentration of taurocholate, a primary bile acid, induced biofilm formation, PirAB*^vp^* toxin release and facilitated the dispersion of bacterial cells. Taken together, our findings suggest that AHPND infection can affect the lipid metabolites in shrimp stomach, and further suggest that the primary bile acid taurocholate is important for the virulence of AHPND-causing *V. parahaemolyticus*.

## Introduction

In recent decades, growing demands for shrimp have transformed the industry from traditional farming into widespread commercial production (Walker and Mohan, 2009). However, frequent outbreaks of viral and bacterial diseases have caused extensive production losses in global shrimp aquaculture (Marvasti and Carter, 2016). Since 2009, many Southeast Asian countries have been affected by outbreaks of acute hepatopancreatic necrosis disease (AHPND), a bacterial disease caused by virulent strains of *Vibrio parahaemolyticus* (Tran et al., 2013; Shinn et al., 2018; Kumar et al., 2020b). The AHPND-causing *V. parahaemolyticus* is a virulent pathogen that affects white shrimp (*Litopenaeus vannamei*), causing high mortality (Tran et al., 2013; Lai et al., 2015; Lee et al., 2015). Its pathogenesis is associated with a unique plasmid (pVA1) that harbors the binary PirA*^vp^* and PirB*^vp^* pore-forming toxins (Lee et al., 2015). It has already been shown that AHPND-causing bacteria initially colonize the shrimp stomach; the binary PirA*^vp^* and PirB*^vp^* toxins produced by these pathogenic bacteria are then released from stomach into the hepatopancreas, causing necrosis of the epithelial cells and infiltration of hemocytes into the hepatopancreas (Lai et al., 2015; Ng et al., 2018; Kumar et al., 2020a).

Shrimp microbiome studies related to shrimp health and disease are increasing (Chen et al., 2017; Yao et al., 2018; Yu et al., 2018). Microbiome refers to bacterial communities associated with a host system. Microbiome function and composition are linked to host health; colonization, growth, and virulence of invading pathogens affect the host system and influence its microbiome (Sassone-Corsi and Raffatellu, 2015; Bäumler and Sperandio, 2016). *Vibrio* spp. use environmental nutrients and cause gastrointestinal diseases in aquatic animals (Johnson, 2013; De Schryver et al., 2014). It has been found that dysbiosis in shrimp stomach occurs during AHPND infection (Chen et al., 2017). Similarly, we also determined that virulent *Vibrio* spp. may alter nutrient availability for gut bacteria and affect host metabolic functions (unpublished data). Metabolomics profiling, an emerging technique in aquaculture, may provide insights into metabolic pathways altered during AHPND pathogenesis.

In general, bacterial pathogens co-evolve with their host and develop mechanisms to respond or resist the host’s environment (Sistrunk et al., 2016). To establish and survive in the host gastrointestinal system, enteric pathogens have to overcome harsh conditions e.g., low pH, immune responses, bactericidal effects of bile acids (BAs), and low nutrient availability (Sistrunk et al., 2016; Lustri et al., 2017). Bile acids are composed of bile salts, fatty acids, and inorganic salts that aid lipid metabolism and have antibacterial properties (Merritt and Donaldson, 2009). Enteric pathogens not only survive these conditions, but they have also evolved mechanisms to resist actions of bile acids and to effectively colonize and infect their host (Merritt and Donaldson, 2009). We reported that crude bile acids affected AHPND-causing *V. parahaemolyticus* by facilitating biofilm formation and stimulating increased release of PirAB*^vp^* toxins (Kumar et al., 2020b). Bile acids are composed of primary and secondary bile salts; in *Vibrio cholerae*, individual bile components had differential effects on biofilm formation and regulation of virulence (Hay and Zhu, 2015; Sanchez et al., 2016). In recent years, bile acids or individual bile components (e.g., taurocholate) are widely used as feed supplements in aquaculture (Yue et al., 2012; Daniel, 2016; Gu et al., 2017).

In this study, we applied a systems biology approach to detect important pathway(s) in the AHPND-affected shrimp stomach metabolome. Metabolomic profiling of AHPND-affected shrimp stomach confirmed the presence of distinct metabolites. These AHPND-associated metabolites were then annotated to identify several dysregulated metabolic pathways, with lipid metabolism and primary bile acid synthesis majorly affected. In the primary bile acid synthesis pathway, metabolites taurocholate and taurochenodeoxycholate were downregulated during AHPND infection. Hence, we investigated the effects of taurocholate on biofilm formation, PirAB*^vp^* toxin release and biofilm detachment in AHPND-causing *V. parahaemolyticus*.

## Materials and Methods

### Experimental Shrimp

*Litopenaeus vannamei* shrimp (average ~1.0–1.5 g) were purchased from the International Center for the Scientific Development of Shrimp Aquaculture, National Cheng Kung University (NCKU) and the Department of Aquaculture, National Pingtung University of Science and Technology (NPUST). Shrimps were selected for their vigor and hard-shell excellence and kept in 30 ppt sterilized seawater for 1–3 days at 26°C until immersion challenge.

### Bacterial Strains

In this study, the following bacterial strains were used for *in vivo* or *in vitro* assays. The *V. parahaemolyticus* AHPND-causing strain (5HP), the non-AHPND causing strain (S02), and the mild AHPND-causing strain (M1-1) were used and cultured at 30°C in Tryptic soy broth (TSB) with 2% sodium chloride (NaCl). In addition, for biofilm assays, *Escherichia coli* and *Staphylococcus aureus* were grown overnight at 37°C in Luria-Bertani (LB) broth.

### Immersion Challenge Test and AHPND Detection

Immersion challenge was performed as described previously (Lai et al., 2015; Ng et al., 2018; Kumar et al., 2020a). Briefly, *V. parahaemolyticus* bacterial strains (5HP or S02) were cultured overnight in TSB medium containing 2% NaCl and growth assessed by measuring optical density (OD) at 600 nm to obtain a concentration of 10^7^ CFU/ml. For immersion challenge, each culture (~100 ml) was mixed with seawater (900 ml). Shrimp were immersed in the bacteria-seawater mixture for 15 min and then transferred back to their respective tanks. In addition, 300 ml of immersion mix was added into the tank containing 30 L of seawater (bacterial density of 10^4^ CFU/ml). This challenge dosage has previously been shown to result in AHPND lesions, i.e., hepatopancreatic epithelium necrosis, within 3 hours post infection (hpi) and to induce high mortality between 12 and 24 hpi (Ng et al., 2018). Stomach samples were collected at 12 and 24 h post immersion. For AHPND detection, shrimp stomach DNAs were extracted as described before (Kumar et al., 2020a) and screened for the presence of AHPND-causing bacteria using an IQ2000 AHPND/EMS Toxin 1 Detection and Prevention System (Gene Reach Biotechnology Corp.).

### Metabolomics: Sample Preparation and Analysis

Sample preparation of shrimp stomach samples collected at 12 hpi and the analysis were done as described (Kumar et al., 2020a). Briefly, 20 μl of internal standard were added to 50 mg of stomach samples and processed with 1 ml of extraction liquid (methanol: acetonitrile: water = 2:2:1). Tissue samples were homogenized using a ball mill and ultrasonicator and then stored at −20°C to precipitate proteins. The supernatant (S; 500 μl) was then aspirated to a fresh tube and extracts were dried using vacuum concentrator. The mixture was sonicated and centrifuged (15,000 × *g*, 15 min, 4°C) and then the S (60 μl) was aspirated to a new liquid chromatography (LC)/mass spectrometry (MS) glass vial, with 10 μl from each sample (shrimp *n* = 6) was pooled with quality control (QC) samples and ~60 μl of sample used for ultra-high-performance liquid chromatography (UHPLC)-quadrupole time-of-flight (QTOF)-MS analysis.

Liquid chromatography/mass spectrometry/mass spectrometry analyses were carried out using an UHPLC system (Agilent Technologies, United States) coupled with TripleTOF 6600 (Q-TOF, AB Sciex). Metabolites were analyzed in both positive (POS) and negative (NEG) ion modes to obtain maximum ionization and reduce background noise, respectively. MS/MS spectra were obtained from Triple TOF mass spectrometer on an Information Dependent Acquisition (IDA). MS/MS spectra data were collected using acquisition software (Analyst TF 1.7, AB Sciex).

### Data Pre-processing and Annotation

ProteoWizard was used to convert MS raw data (.d) to the mzXML format and then data were processed by R package XCMS (Ver. 3.2). Unprocessed data produced a data matrix of peak intensity, mass-to-charge ratio (m/z) values, and retention time (RT). Processed data peaks from XCMS were further analyzed using R-package Collection of Algorithms for MEtabolite pRofile Annotation (CAMERA) for peaks, adducts, and fragment annotations in the LC-MS data (Kuhl et al., 2012). The cutoff for match score was set as 0.6 and the minfrac was set as 0.5. Annotated metabolites were identified using a proprietary in-house MS1 and MS2 database (Biotree Tech., Shanghai, China).

### Gene Expression Analysis

Total RNA from shrimp stomach samples collected at 12 and 24 hpi were extracted using Rezol C&T reagent and complementary DNA (cDNA) synthesized using Superscriptase II Reverse Transcriptase (Invitrogen). Using specific primers real-time PCR was performed on a Bio-Rad detection system with KAPA SYBR1 FAST Master Mix (KAPA) to determine relative gene expression of host genes. Primer sequences to identify bile acid synthesis genes were as described (Kumar et al., 2020a). Genes involved in unsaturated fatty acid biosynthesis and bile acid biosynthesis were identified from our in-house AHPND-affected shrimp stomach transcriptomic database (Ng et al., 2018) and primer sequences are shown in Supplementary Table S1. Data were normalized to elongation factor 1α (EF1α; housekeeping gene) and the relative expression was calculated by the 2^-ΔΔCT^ method.

### Quantification of Biofilm Formation by Crystal Violet Staining

The biofilm formation assay was performed as described in Kumar et al. (2020a). Briefly, *E. coli* and *S. aureus* were cultured in LB broth overnight at 37°C and the M1-1, S02, 5HP were grown in TSB+2% NaCl overnight at 30°C. Cultures were grown and adjusted to OD_600_ = 0.6, diluted (1:100) and 100 μl were added to 96-well plates containing various concentrations of taurocholate. The 0.25% bile acid and samples without bile acid or taurocholate (no-bile-acid) were used as controls. Sodium taurocholate was procured from Biosynth Carbosynth (CAS no. 145-42-6) and a 100 mM stock solution was prepared with water. To achieve a concentration range similar to that described in Sanchez et al. (2016), the taurocholate was serially diluted with TSB medium to obtain concentrations of 0.03, 0.06, 0.12, 0.5, 1, and 2 mM. The 96-well plates were incubated at 30 and 37°C, respectively, for 24 h at static conditions. Cells were then washed, stained with 0.1% crystal violet and incubated for 15 min at room temperature. Then, cells were washed with phosphate-buffered saline (PBS) and biofilms were solubilized in 30% acetic acid and incubated for 15 min at room temperature. An aliquot (100 μl) of solubilized biofilm was pipetted to a fresh 96-well plate and measured the absorbance at 550 nm. Samples were analyzed as five replicates and data were normalized to blank (30% acetic acid) and control (no-bile-acid) samples. Biofilm data were represented as relative biofilm formation compared to the no-bile-acid group, with error bars indicating SD. The statistically significant difference between control and treatment groups were calculated using Student’s *t*-test (^*^p < 0.05, ^**^p < 0.01, and ^***^p < 0.001).

### Measurement of the Number of Planktonic and Attached AHPND-Causing *Vibrio parahaemolyticus* 5HP From the Biofilm Assay

An overnight culture of 5HP was diluted (1:100) and 100 μl of culture was incubated with 0.25% crude bile acid and various concentrations of taurocholate (0.03, 0.06, 0.12, 0.5, 1, and 2 mM) in a 96-well plate, with no-bile-acid used as a control. The plate was then incubated at 30°C for 24 h at static conditions. Concurrently, the 1:100 diluted 5HP culture was further serially diluted (10^1^–10^6^), and 100 μl of 10^6^ dilution was plated on thiosulfate citrate bile salts sucrose (TCBS) agar in duplicates. After incubating plates overnight at 30°C, colonies were enumerated and colony-forming unit (CFU) calculated [CFU/ml = (Number of colonies × dilution factor)/volume of culture]. To confirm biofilm formation, after 24 h of incubation, a portion of the biofilms from the 96-well plate was stained using 0.1% crystal violet (as described earlier). From the remaining biofilms, the supernatant was carefully aspirated into a new 1.5 ml tube. The supernatants and harvested biofilms were further serially diluted (10^1^–10^6^) and 100 μl of 10^6^ dilution was plated on TCBS agar in duplicates. The TCBS agar plates were incubated at 30°C for 16 h, enumerated colonies and calculated CFU.

### Western Blot Analysis of PirAB*^vp^* Toxins

Western blot was performed as described in Kumar et al. (2020a). In brief, an overnight culture of 5HP was adjusted to OD_600_ = 1.5, diluted (1:100) with TSB medium, and added to tubes containing taurocholate to achieve 1.5 ml aliquots with final taurocholate concentrations of 0.007, 0.03, 0.06, 0.12, 0.5, 1, and 2 mM. At 3 and 16 h, cells were harvested by centrifugation (8,000 × *g* for 10 min) at 4°C. Bacteria pellets (P) were resuspended with 1.5 ml PBS, and 10 μl of the P suspension and supernatant were separately run on 15% sodium dodecyl sulfate polyacrylamide gel electrophoresis (SDS-PAGE) and transferred onto a polyvinylidene fluoride (PVDF) membrane. Membranes were initially probed with primary antibodies for PirB*^vp^* (~51 kDa) and PirA*^vp^* (~12 kDa) in 2% bovine serum albumin (BSA) in Tris buffered saline tween-20 (TBST) for 1 h at room temperature. Then, blots were stained with secondary antibody conjugated with horseradish peroxidase (HRP). Signals were detected using a luminol-based Western Lightning Plus-ECL reagent kit (Perkin Elmer) and the intensities were detected using an ImageQuant LAS 4000 mini (GE Healthcare Life Science). ImageJ software (NIH, Bethesda, MD, United States) was used to quantify intensity of bands in immunoblots.

## Results

### Identification of Differentially Expressed Metabolites Associated With AHPND

Stomach samples were collected from shrimp challenged with 5HP or S02, and TSB control. Stomach samples from the 5HP-infected group were tested for expression of pVA plasmid and PirAB*^vp^* toxins (data not shown). The AHPND-affected samples, along with their corresponding controls (*n* = 6, 4 stomachs/pool) were pooled and sent for UHPLC-QTOF-MS-based metabolomics.

**Figure 1:**
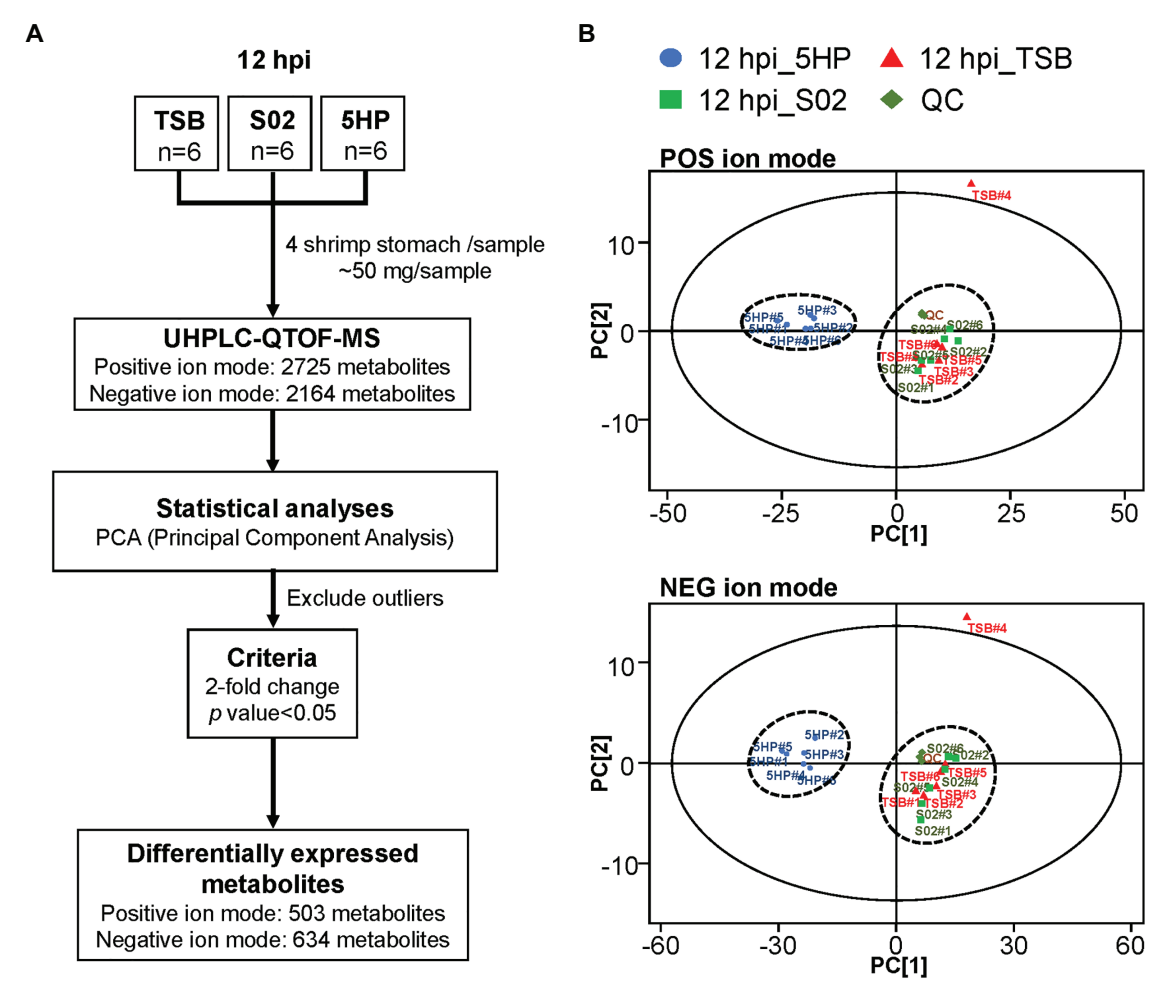
Overview of metabolome profiling assembly pipeline and principal component analysis (PCA) of differentially expressed metabolites (DEMs). **(A)** Acute hepatopancreatic necrosis disease (AHPND)-causing strain (5HP)-infected shrimp stomach samples were used for metabolomic profiling using UHPLC-QTOF-MS. In total, 2,725 and 2,614 metabolites were obtained in positive (POS) and negative (NEG) ion modes, respectively. **(B)** Principle component analysis of DEMs from samples collected from quality control (QC), tryptic soy broth (TSB), non-AHPND-causing strain (S02), and 5HP groups. The 18 pooled samples and three QCs analyzed in this study were classified into two distinct clusters in both POS and NEG ion modes. The PCA analysis also identified TSB#4 as an outlier; this sample was excluded from further analyses.

**Figure 2:**
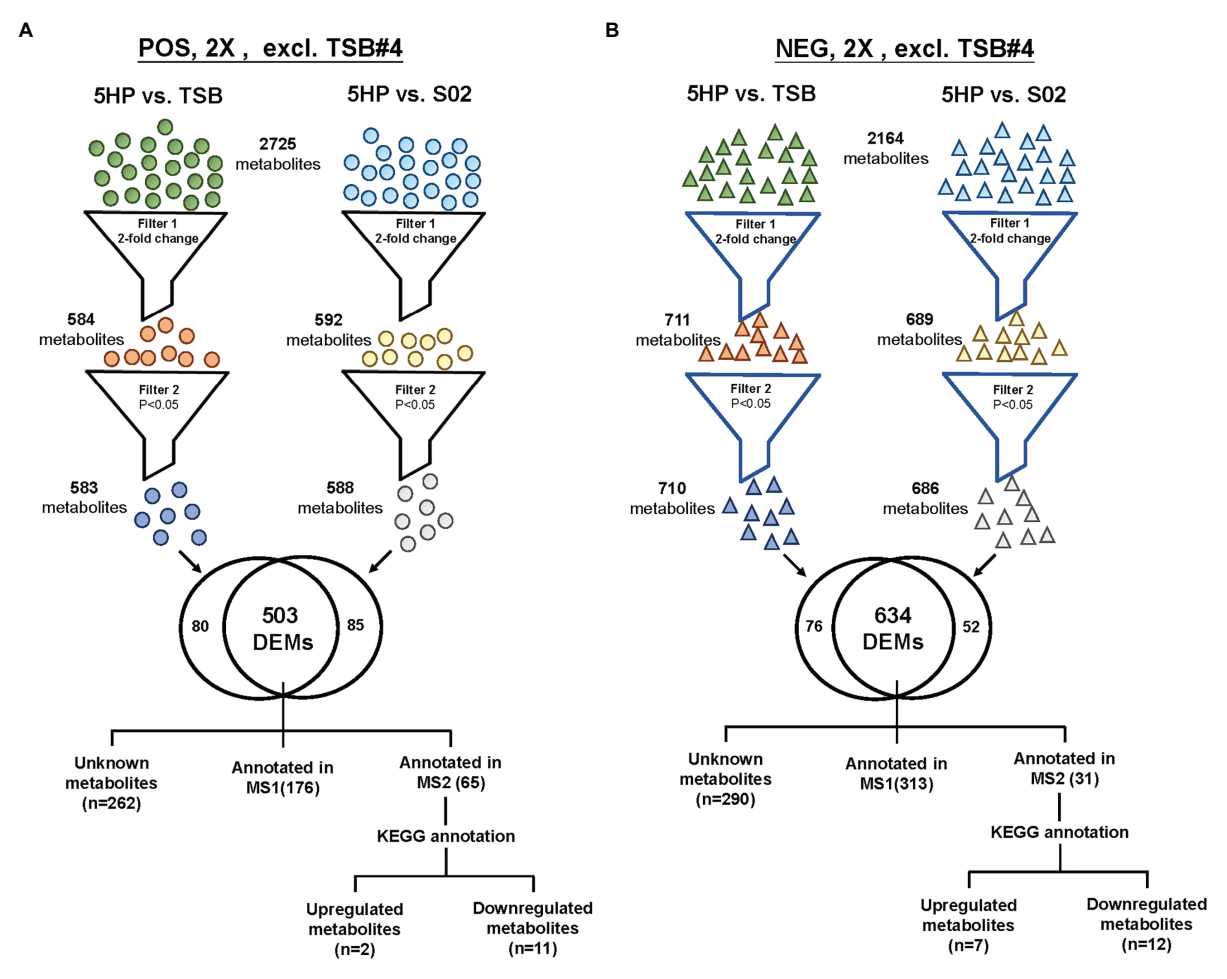
The strategy of data filtering for shrimp metabolites. Comparison of DEMs in both POS ion and NEG ion modes of 5HP vs. TSB and 5HP vs. S02 at 12 hours post infection (hpi). **(A)** In POS ion mode, from the 2,725 metabolites, there was at least one 5HP, TSB, or S02, 503 DEMs with a 2-fold change (*p* < 0.05). **(B)** Similarly, from the 2,164 metabolites in NEG ion mode, 634 DEMs with a 2-fold change and *p* < 0.05 were obtained. The DEMs were further annotated using proprietary MS1, MS2 database, and Kyoto encyclopedia of genes and genomes (KEGG).

An overview of the metabolomics profiling analysis pipeline is shown (Figure 1A). There were 2,725 and 2,164 metabolites obtained in POS and NEG ion modes, respectively. In principal component analysis (PCA) analysis (Figure 1B), distinct clusters formed by the metabolites of 5HP-infected shrimp samples in both POS and NEG ion modes implied that composition of the metabolites in the 5HP-infected group was different from that of the S02 and TSB controls. To further identify which metabolites were differentially expressed in the 5HP-infected group, data were filtered to include only metabolites with >2-fold change and *p* < 0.05. In the POS mode, 5HP vs. TSB yielded 584 metabolites with ≥2X fold change and 583 metabolites with *p* < 0.05, whereas 5HP vs. S02 generated 592 metabolites with ≥2X fold change and 588 metabolites with *p* < 0.05, for a total of 503 differentially expressed metabolites (DEMs; Figure 2A). Similarly, 634 DEMs were identified in NEG ion mode (Figure 2B). A similar analysis of S02 vs. TSB yielded no DEMs (data not shown), implying that the S02-challenged group had no effect on the composition of the metabolites in shrimp stomach compared to the TSB control.

**Figure 3:**
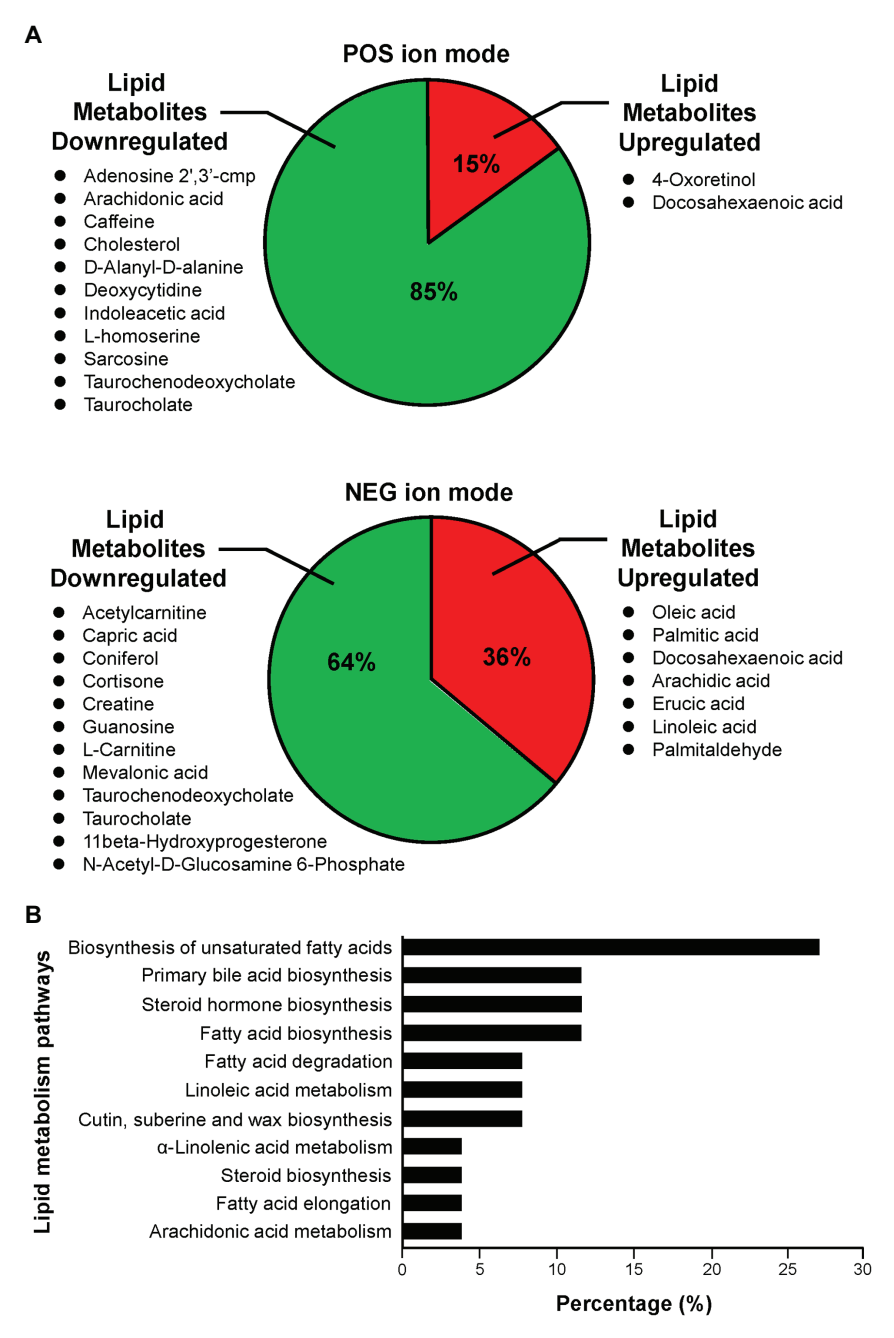
Functional classification of DEMs according to their corresponding pathways. **(A)** Proportion of upregulated and downregulated metabolites associated with lipid metabolism of 5HP-infected shrimps in POS and NEG ion modes. **(B)** KEGG pathway breakdown of the lipid metabolism DEMs. Metabolites without MS/MS annotations were excluded.

In the 5HP-infected group, the POS and NEG DEMs were then annotated to two proprietary databases MS1 and MS2, followed by Kyoto Encyclopedia of Genes and Genomes (KEGG) annotation. This yielded two upregulated metabolites and 11 downregulated metabolites in POS ion mode (Supplementary Table S2). In NEG ion mode, seven DEMs were upregulated and 12 DEMs were downregulated (Supplementary Table S3). In POS ion mode, 15% metabolites were upregulated, whereas 85% were downregulated (Figure 3A). In the NEG ion mode, 36% metabolites were upregulated and 64% were downregulated. Interestingly, in both POS and NEG ion modes, bile acid components taurocholate and taurochenodeoxycholate were downregulated (Figure 3A). To further investigate key lipid metabolism pathways of these metabolites, functional classification of specific DEMs of 5HP-infected group stomach metabolome were annotated using KEGG pathway analysis. By percentage of annotations, the top four metabolic pathways were: biosynthesis of unsaturated fatty acids, primary bile acid biosynthesis, steroid hormone biosynthesis, and fatty acid biosynthesis (Figure 3B).

### AHPND Upregulated Expression of Genes Involved in the Biosynthesis of Unsaturated Fatty Acids

**Figure 4:**
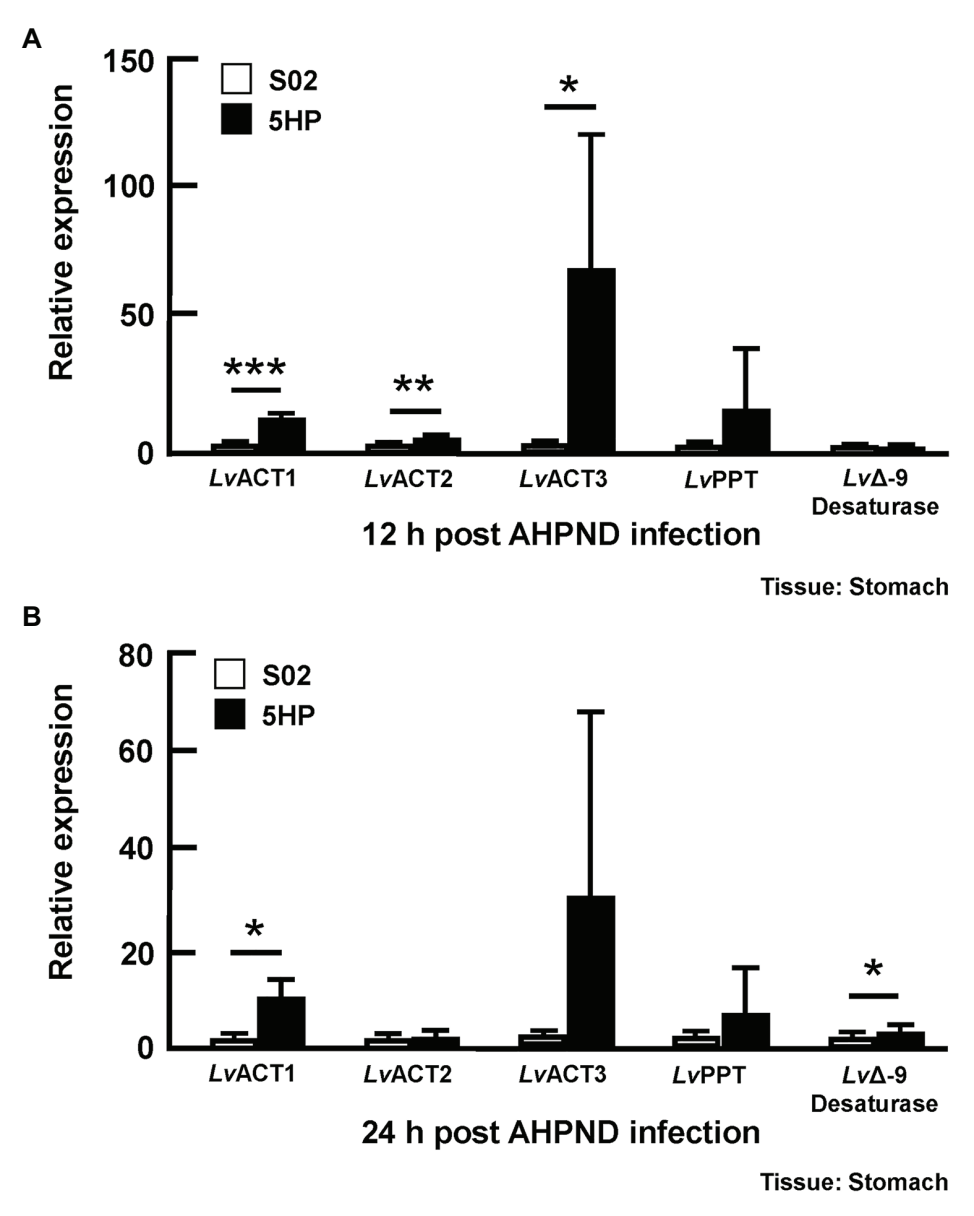
Acute hepatopancreatic necrosis disease infection affected the expression of genes related to the biosynthesis of unsaturated fatty acids. Stomach samples of 5HP-challenged and S02-challenged shrimps were collected at **(A)** 12 and **(B)** 24 hpi were analyzed for expression of genes involved in the biosynthesis of unsaturated fatty acids (*Lv*ACT: Acyl-coenzyme A thioesterase, *Lv*PPT: Palmitoyl-protein thioesterase, *LvΔ*-9 Desaturase). Graphs represent mean ± SD. Differences between treatment groups are indicated by asterisks (Student’s *t*-test, ^*^p < 0.05, ^**^p < 0.01, and ^***^p < 0.001).

To further investigate whether genes involved in the biosynthesis of unsaturated fatty acid were affected during AHPND infection, the expression of several genes in this pathway [Acyl-coenzyme A thioesterase (*LvACT1*, *LvACT2*, *LvACT3*; EC 3.1.2.20), Palmitoyl-protein thioesterase (*LvPPT*; EC 3.1.2.22), and stearoyl-CoA desaturase (*LvΔ*-9 desaturase; EC 1.14.19.1) was determined in shrimp that had been confirmed to be infected with AHPND (Supplementary Figure S1). At 12 hpi, compared to the S02-challenged group, the expression of *LvACT1*, *LvACT2*, and *LvACT3* was significantly induced in 5HP-infected shrimp (Figure 4A), while at 24 hpi, only *LvACT1* was significantly induced (Figure 4B). No significant change in the expression of *LvPPT* was found between the S02-challenged group and 5HP-infected group at 12 or 24 hpi (Figure 4). For the *Lv*Δ-9 desaturase, there was only a significant increase in expression at 24 hpi (Figure 4). Overall, except for *Lv*PPT, all the tested genes involved in the biosynthesis of unsaturated fatty acids showed some significant upregulation during AHPND infection.

### AHPND Induced the Expression of Genes Involved in Bile Acid Synthesis at 24 hpi

**Figure 5:**
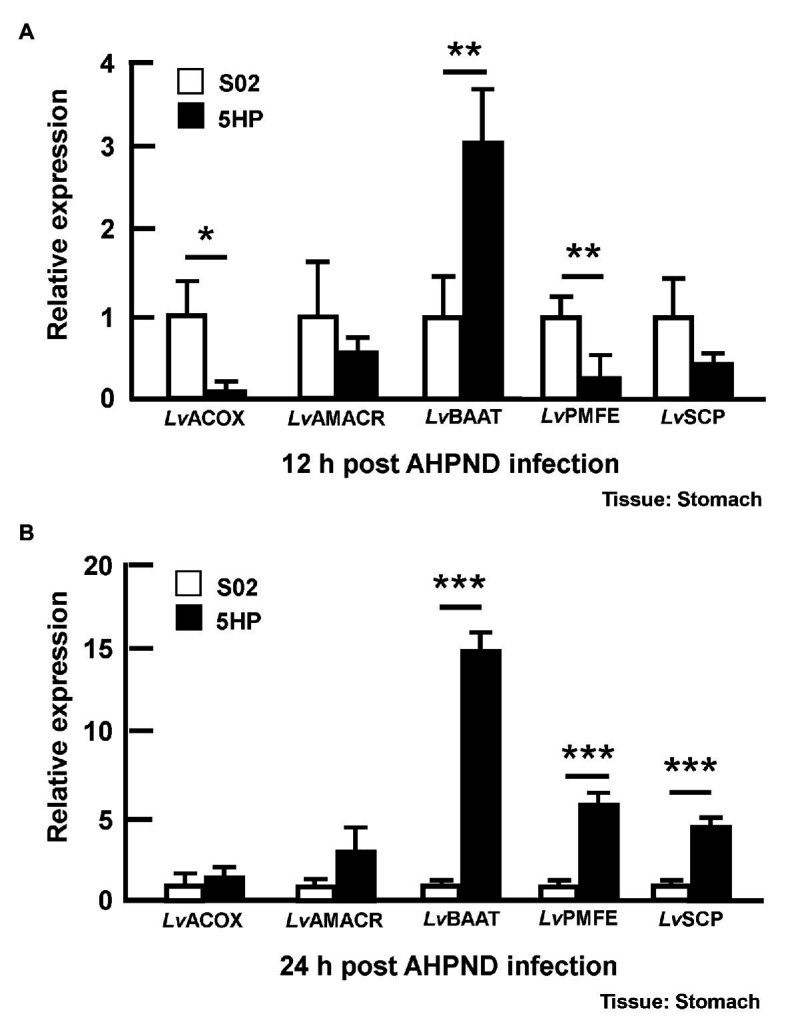
Expression of bile acid synthesis genes was modulated during AHPND infection. Stomach samples collected at **(A)** 12 and **(B)** 24 hpi from shrimp challenged with either S02 or 5HP were used to analyze gene expression of bile acid synthesis related genes (*Lv*ACOX, *Lv*AMACR, *Lv*BAAT, *Lv*PMFE, and *Lv*SCP). Data are mean ± SD. Differences between treatment groups are indicated by asterisks (Student’s *t*-test, ^*^p < 0.05, ^**^p < 0.01, and ^***^p < 0.001).

Since the bile acid metabolites were altered during AHPND infection (Figure 3A), we also examined the expression of the bile acid synthesis genes in stomach samples taken from the same S02-challenged and 5HP-infected shrimp (please see Supplementary Figure S1) at 12 and 24 hpi. Several genes involved in the bile acid synthesis were identified previously (Kumar et al., 2020a), namely Alpha-methylacyl-CoA racemase (*LvAMACR*; EC 5.1.99.4), Acyl-coenzyme A oxidase (*LvACOX*; EC 1.3.3.6), Bile acid acyl-coenzyme A thioesterase (*LvBAAT*; EC2.3.1.65), Sterol carrier protein (*LvSCP*), and Peroxisomal multifunctional enzyme (*LvPMFE*; EC4.2.1.119). The *LvACOX* gene was significantly downregulated at 12 hpi (Figure 5A), whereas at 24 hpi, there was no change in the expression compared to the S02-challenged group (Figure 5B). The *LvBAAT* gene expression was significantly upregulated at both 12 and 24 hpi in 5HP-infected groups (Figures 5A,B). At 12 hpi, the *LvPMFE* expression was significantly downregulated (Figure 5A), whereas at 24 hpi it was significantly upregulated compared to the S02-challenged group (Figure 5B). Similarly, the *LvSCP* did not have any change in expression at 12 hpi (Figure 5A), whereas at 24 hpi, it was significantly upregulated in the 5HP-infected group (Figure 5B). We inferred that AHPND affected the expression of genes involved in bile acid synthesis.

### Taurocholate Induces Biofilm Formation in AHPND-Causing *Vibrio parahaemolyticus*

**Figure 6:**
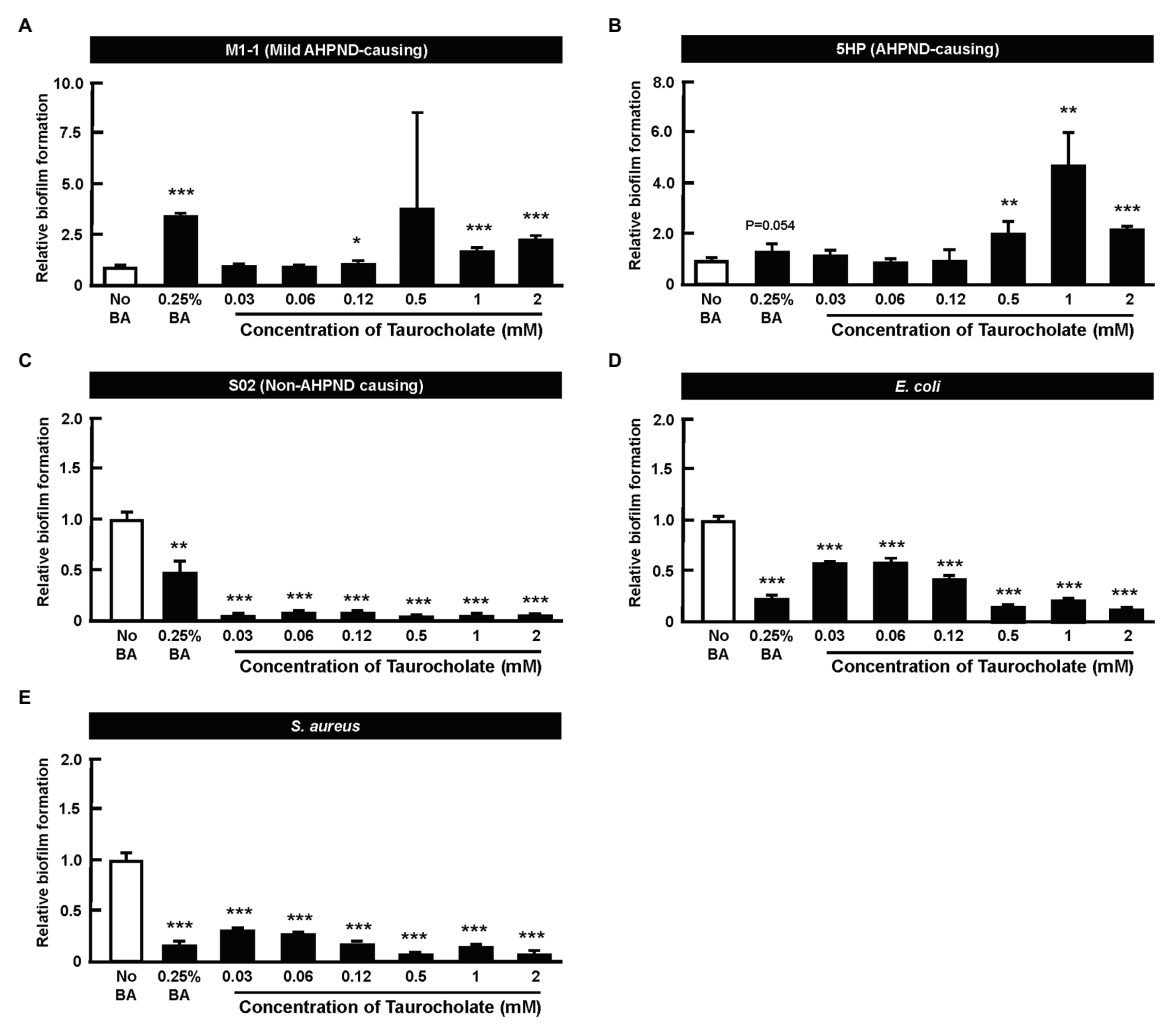
Taurocholate induced biofilm formation in AHPND-causing *Vibrio parahaemolyticus*. Biofilm assays were conducted at various concentrations of taurocholate for **(A)** M1-1, **(B)** 5HP, **(C)** S02, **(D)** *Escherichia coli*, and **(E)** *Staphylococcus aureus* in 96-well plates. The biofilms were stained with crystal violet stain and quantified at OD_550_nm. The data represent fold change compared to no-bile-acid control. Each bar represents mean ± SD. The statistically significant differences between treatment and control groups are indicated as asterisks (Student’s *t*-test, ^*^p < 0.05, ^**^p < 0.01, and ^***^p < 0.001).

A quantitative *in vitro* biofilm formation assay was performed at various concentrations of taurocholate, with no-bile-acid group and the 0.25% crude bile acid treated group. M1-1, 5HP, S02, *E. coli*, and *S. aureus* bacteria were used (Figures 6A–E). In M1-1 and 5HP strain, compared to the no-bile-acid control, lower doses (0.03–0.12 mM) of taurocholate (Figures 6A,B) did not form biofilms, whereas higher concentrations (0.5–2 mM) of taurocholate increased biofilm formation compared to their respective no-bile-acid control. However, *E. coli*, *S. aureus*, and S02 did not show biofilm formation with or without taurocholate (Figures 6C–E).

### Taurocholate Also Increases the Number of Planktonic AHPND-Causing *Vibrio parahaemolyticus* in Cultured Supernatant

**Figure 7:**
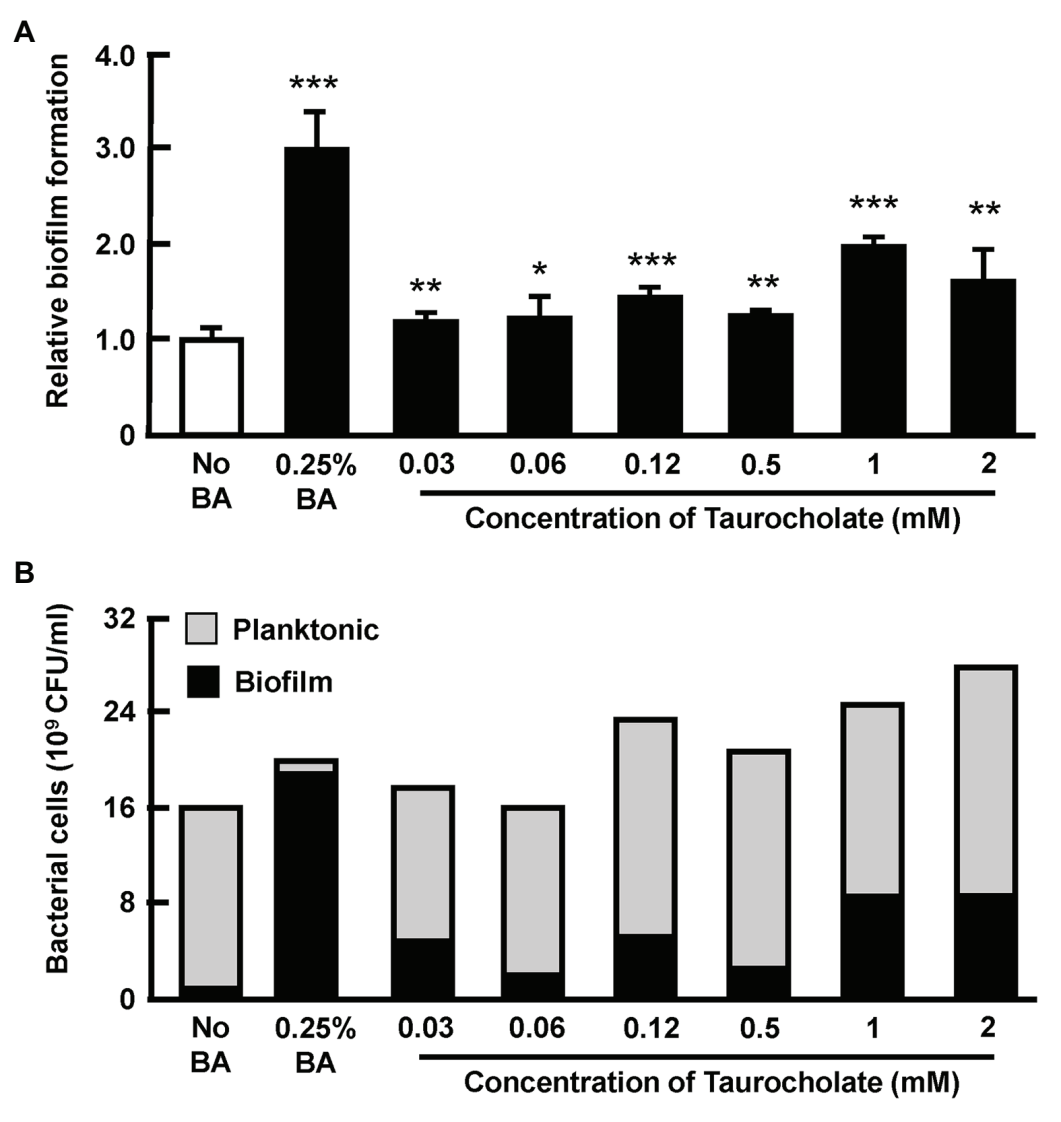
Taurocholate increased the number of both biofilm and planktonic AHPND-causing *V. parahaemolyticus* 5HP in culture supernatant. Effect of taurocholate on the number of planktonic bacterial cells was assessed by treating the AHPND-causing *V. parahaemolyticus* 5HP with crude bile acids and taurocholate. **(A)** Biofilm formation in the presence of taurocholate.The data represent fold change compared to no-bile-acid control. Each bar represents mean ± SD. The statistically significant differences between the treatment groups and the no-bile-acid control group are indicated as asterisks (Student’s t-test, ^*^p < 0.05, ^**^p < 0.01, and ^***^p < 0.001). **(B)** Proportion of planktonic and biofilm cells.

In this assay, cultures of AHPND-causing bacteria 5HP were again treated with various concentrations of taurocholate, and a crystal violet assay showed that compared to the no-bile-acid control, the crude bile acid treated group and the taurocholate-treated groups all showed increased biofilm formation (Figure 7A). A count of the number of bacterial cells further showed that in the crude bile acid treated group, the number of planktonic (non-attached) cells was less than ~5% of the total population, while groups treated with various concentrations of taurocholate had a much higher proportion of planktonic bacteria (~65–87%; Figure 7B). Compared to the no-bile-acid group, the absolute number of planktonic cells was also increased when cells were treated with the higher taurocholate concentrations (0.12~2 mM). We therefore concluded that in addition to increasing biofilm formation (Figure 6), higher concentrations of taurocholate also had a positive effect on the number of planktonic AHPND-causing *V. parahaemolyticus*.

### Taurocholate Increases the Release of PirAB*^vp^* Toxins

**Figure 8:**
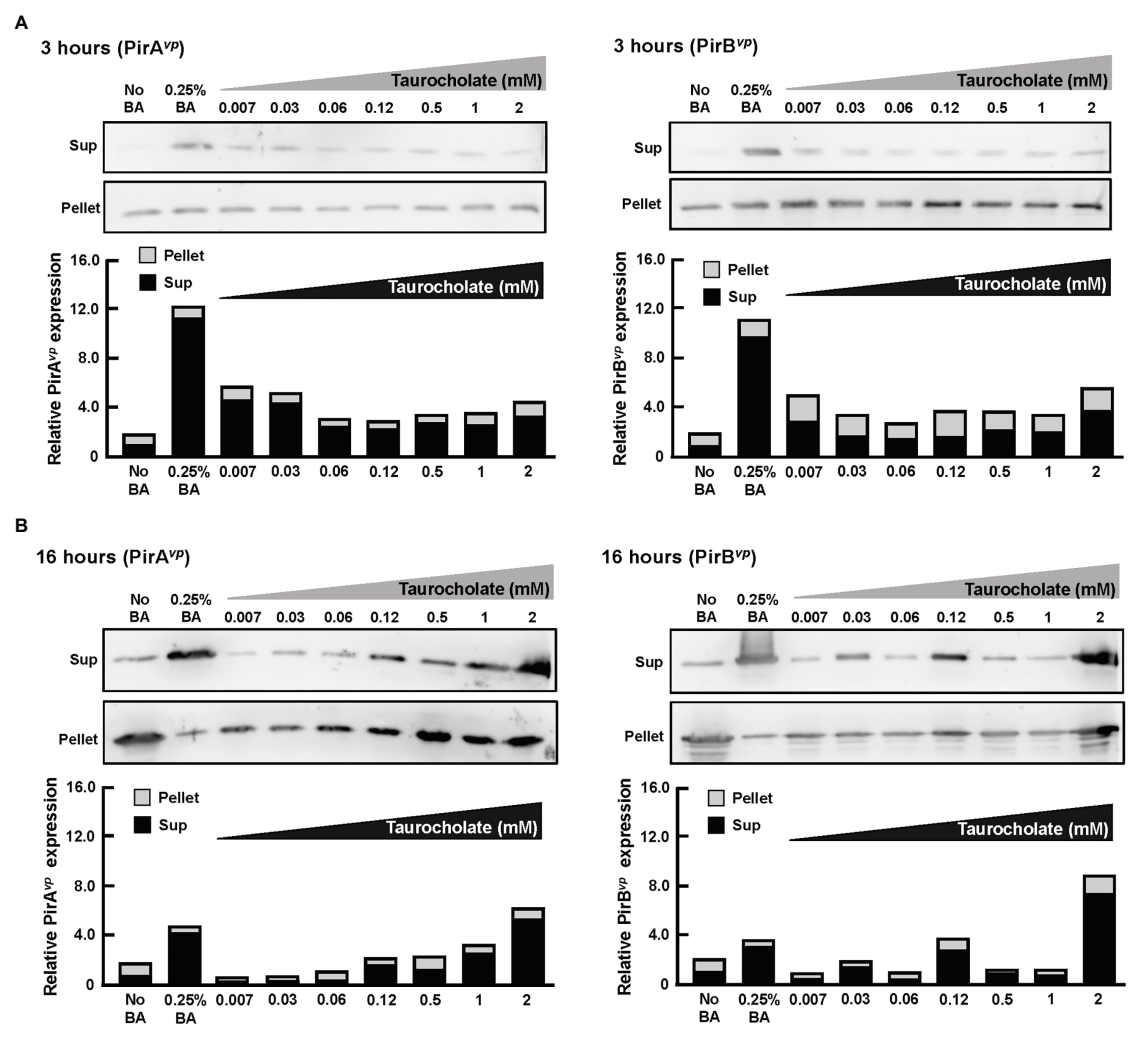
Taurocholate stimulated PirAB*^vp^* toxin release in AHPND-causing *V. parahaemolyticus*. Effects of taurocholate on release of PirA*^vp^* and PirB*^vp^* toxins were assessed by Western blots. Toxin concentrations were detected in the pellet (P) and supernatant (S) of AHPND-causing *V. parahaemolyticus* 5HP strain cultured with taurocholate for **(A)** 3 or **(B)** 16 h. Immunoblots were probed with PirA*^vp^* and PirB*^vp^* antibodies, respectively. The intensities of each pair of immunoblots are shown graphically as relative expression in supernatant (Sup) and pellet compared to their corresponding no-bile-acid control.

Overnight cultures of AHPND-causing *V. parahaemolyticus* strain (5HP) were treated with various concentrations of taurocholate for 3 or 16 h. P and S samples were collected, subjected to Western blotting and probed with antibodies against PirA*^vp^* and PirB*^vp^*. At 3 h, both PirA*^vp^* and PirB*^vp^* were detected in the pellet and supernatant of taurocholate-treated groups (Figure 8A), but no toxins were detected in the supernatant of no-bile-acid control. Interestingly, at 16 h, samples treated with lower concentrations (0.007–0.06 mM) of taurocholate showed PirA*^vp^* toxin levels similar to the no-bile-acid supernatant, whereas higher concentrations (0.5–2 mM) of taurocholate increased toxin concentrations in the supernatant (Figure 8B) compared to the supernatant of no-bile-acid control. However, for PirB*^vp^* toxins, increased the release of toxins into supernatant was seen at specific concentration of taurocholate (0.03, 0.12, and 2 mM; Figure 8B). Relative expression of supernatant to pellet expression ratio of PirA*^vp^* and PirB*^vp^* toxins were calculated by quantifying intensities (see graphs below individual blots). We concluded that taurocholate increased the secretion of PirAB*^vp^* toxins into the extracellular environment in AHPND-causing *V. parahaemolyticus* 5HP.

## Discussion

We have previously reported AHPND altered the composition of bacterial communities in shrimp stomach (Chen et al., 2017). Based on our sequencing data analysis by Phylogenetic Investigation of Communities by Reconstruction of Unobserved States (PICRUSt) and identification of relative KEGG pathway abundance, bacterial metabolic functions may be dysregulated in the AHPND-affected shrimp stomachs (unpublished data). Microbiota dysbiosis altered nutrient availability to gut bacteria and impacted their metabolic functions in gut-associated diseases (Carding et al., 2015; Peterson et al., 2015; Scher et al., 2015). Recent advances in mass spectrometry facilitated quantitative detection of several metabolites from minimal amounts of biological samples (Dettmer et al., 2007). Profiling global metabolites could provide insights into biological mechanisms and cellular pathways that can reveal new discoveries and effective treatment strategies (Fiehn, 2002; Patti et al., 2012).

In this study, we characterized the alterations in metabolites of the 5HP-infected shrimp stomachs. Metabolites were extracted from shrimp stomach and analyzed using UHPLC-QTOF-MS in both POS and NEG ion modes (Figure 1A). Based on PCA analysis of metabolites across treatment groups, metabolites of the 5HP-infected group clustered distinctly compared to the control groups (Figure 1B). We further found that, in KEGG annotation of these DEMs, lipid metabolites were majorly affected (Figure 3A). Key pathways altered during AHPND infection were: biosynthesis of unsaturated fatty acids, primary bile acid biosynthesis, steroid hormone biosynthesis, and fatty acid biosynthesis (Figure 3B). This finding is similar to those seen in the *Vibrio* pathogenesis in drosophila, which also usually causes changes in lipid metabolism (Vanhove et al., 2017; Davoodi and Foley, 2020).

Figure 4 shows that there was an increase in the expression of genes involved in the biosynthesis of unsaturated fatty acids, that is the acyl-coenzyme A thioesterases *LvACT1*, *LvACT2*, *LvACT3*, which convert acyl-CoAs to free fatty acids (Figure 4). Wall et al. (2017) similarly also found that, after mice were treated with Gram negative bacteria cell wall component lipopolysaccharide (LPS), acyl-coenzyme A thioesterase 7 was induced in the early phase of the inflammatory response, with levels subsequently returning to basal values at 24 h. The functional roles of acyl-coenzyme A thioesterases during inflammation and bacterial infection will need to be further elucidated, but we note that the free fatty acids that they produce are thought to have antibacterial activity (Zheng et al., 2005; Desbois and Smith, 2010).

We also found that AHPND induced the expression of genes involved in bile acid synthesis at 24 hpi (Figure 5). As we suggested previously, crude bile acids have positive effect on the biofilm formation and the secretion of PirAB*^vp^* toxins (Kumar et al., 2020a). In humans, pathogenic bacteria are exposed to a combination of both primary bile acids (cholic acid and its conjugates taurocholic acid (TCA) and glycolic acid) and secondary bile acids (glycodeoxycholic acid, deoxycholic acid, and taurodeoxycholic acid; Begley et al., 2005). Individual components of bile acids can affect enteric pathogens (Bachmann et al., 2015). For example, deoxycholic acid induced virulence gene expression in *Campylobacter jejuni* (Malik-Kale et al., 2008). We have previously reported the presence of primary bile acid metabolites such as taurocholate (a taurine-conjugated form of the primary bile acid), taurochenodeoxycholate, deoxycholic acid tauroursodeoxycholic acid, taurodeoxycholic acid, and taurolithocholic acid in AHPND-affected shrimp stomach (Kumar et al., 2020a). In the present study, we found that higher concentrations (0.5~2 mM) of taurocholate were associated with increased biofilm formation in AHPND-causing *V. parahaemolyticus* (Figure 6B). Curiously however, treatment with bile acids and taurocholate did not induce biofilm formation in the non-AHPND *V. parahaemolyticus* strain S02 (Figure 6C). The reason for this remains unclear, but we note that strain S02 was isolated from shrimp pond sediment prior to any local outbreaks of AHPND (Joshi et al., 2014), and it may have significant genomic differences from strains S02 and 5HP. In *V. cholerae*, TCA was involved in biofilm dispersal, as well as induced virulence by increasing the expression of *tcpA* (Hay and Zhu, 2015). Herein, PirAB*^vp^* toxin was released into the supernatant at 3 h compared to the no-bile-acid control (Figure 8A). However, at 16 h, low taurocholate concentrations stimulated a lesser toxin release, whereas higher concentrations increased the release of PirA*^vp^* toxins into the supernatant (Figure 8B). Although a similar trend was not observed for PirB*^vp^* an increased toxin release was seen at certain concentrations of taurocholate (Figure 8B). The regulation of virulence by bile acids was reported in *V. parahaemolyticus* that causes gastroenteritis in humans: the bacteria respond to the bile acids in their surroundings by regulating their type III secretion system 2 (T3SS2) and releasing toxins (Gotoh et al., 2010; Letchumanan et al., 2017). Similarly, in *V. cholerae*, bile acids have shown to induce the cholera toxin production by regulating various virulence factors (Hung and Mekalanos, 2005).

The bile acids possess antibacterial properties that can disrupt the bacterial cell membrane by targeting the membrane phospholipids and proteins (Letchumanan et al., 2017). Enteric pathogens not only develop bile resistance, but also use bile as a signal to regulate their virulence (Yang et al., 2013). For example, taurocholate treatment increases the release of exopolysaccharides from *V. cholerae* biofilms (Hay and Zhu, 2015). In addition, host factors such as concentrations of bile acids and bicarbonate influence the transition between biofilm and planktonic lifestyles of *V. cholerae* during colonization of human intestines (Silva and Benitez, 2016). Bile acid constituents have selective and distinct effects on biofilm formation in bacterial pathogens (Sanchez et al., 2016). For example, the treatment of *V. cholerae* biofilms with TCA or taurochenodeoxycholic acid (TCDCA) reduced biofilm formation and increased dispersal of cells from the biofilms (Sanchez et al., 2016). Interestingly, in our study, taurocholate also induced an increase in the number of planktonic bacterial cells (Figure 7). In a future study, we hope to investigate whether these detached bacteria might emulate the colonization process of *V. cholerae* (Hay and Zhu, 2015) and subsequently establish secondary colonies of the AHPND-causing *V. parahaemolyticus* in shrimp stomach. To further explore the mechanism by which taurocholate is able to induce 5HP to form biofilm, in the future, we also hope to investigate the effect that different concentrations of taurocholate may have on the morphology and motility of 5HP.

We note that since AHPND infection can alter the composition and structure of the microbial communities in shrimp stomachs and thereby induce microbiota dysbiosis (Chen et al., 2017), it follows that the observed alterations in the metabolic profiles of diseased shrimp stomachs may be also related to these changes in the microbiota. At present, we are unable to fully explore this possibility, but it will be interesting to further investigate the relationship between the host, the AHPND-causing bacteria, and the AHPND-induced dysbiosis.

## Conclusion

In conclusion, we identified several differentially expressed metabolites in the stomach of AHPND-affected shrimp that were used to identify changes in metabolic pathways during AHPND infection. Several genes related to lipid metabolism and primary bile acid synthesis were dysregulated in shrimp stomach during AHPND infection. Taurocholate, a key metabolite, was downregulated in metabolomics data and addition of taurocholate to a culture of AHPND-causing *V. parahaemolyticus* was sufficient to induce biofilm formation, release toxin and facilitate the dispersion of bacterial cells. We hope to continue by exploring the possibility that other primary and secondary bile acids are also involved in AHPND pathogenesis. Meanwhile, the present results should help to elucidate the pathogenesis of AHPND and identify key factors of AHPND infection.

## Data Availability Statement

The original contributions presented in the study are included in the article/Supplementary Material; further inquiries can be directed to the corresponding author.

## Ethics Statement

Ethical review and approval was not required for the animal study because we used shrimp in this study. These animals were specifically raised for research purposes and Taiwan does not require any additional permit or permission. Because the experimental animals were invertebrates, no specific permits were required for this study, and there is no official recommendation for the use of shrimp for scientific purposes in Taiwan. Nevertheless all of our experimental procedures, including animal sacrifice, were designed to be as humane as possible, and all animals were treated so as to minimize suffering at all times.

## Author Contributions

H-CW conceived and designed the experiments. S-SL and Y-MC performed data analysis and bioinformatics. RK, T-CT, Y-LC, and C-CC performed the experiments and analyzed data. RK, T-CT, and H-CW wrote the manuscript. THN was responsible for data coordination and data analysis. All authors contributed to the article and approved the submitted version.

### Conflict of Interest

The authors declare that the research was conducted in the absence of any commercial or financial relationships that could be construed as a potential conflict of interest.

## Figures and Tables

**Table 1:** 

5HP	AHPND-causing strain
ACOX	Acyl-coenzyme A oxidase
ACT	Acyl-coenzyme A thioesterase
AHPND	Acute hepatopancreatic necrosis disease
AMACR	Alpha-methylacyl-CoA racemase
BA	Bile acid
BAAT	Bile acid acyl-coenzyme A thioesterase
BSA	Bovine serum albumin
CAMERA	Collection of algorithms for metabolite profile annotation
cDNA	Complementary DNA
CFU	Colony-forming unit
DEMs	Differentially expressed metabolites
EF1α	Elongation factor 1α
h	Hour
hpi	Hours post infection
HRP	Horseradish peroxidase
IDA	Information dependent acquisition
KEGG	Kyoto encyclopedia of genes and genomes
L	Liter
LB	Luria broth
LC	Liquid chromatography
Lv	*Litopenaeus vannamei*
M1-1	Mild AHPND-causing strain
ml	Milliliter
μl	Microliter
mg	Milligram
MS	Mass spectrometry
m/z	Mass-to-charge ratio
mM	Millimolar
min	Minute
NaCl	Sodium chloride
NEG	Negative
nm	Nanometer
OD	Optical density
P	Pellet
PBS	Phosphate-buffered saline
PCA	Principal component analysis
PCR	Polymerase chain reaction
Pir	*Photorhabdus luminescens* insect-related
PICRUSt	Phylogenetic investigation of communities by reconstruction of unobserved states
PMFE	Peroxisomal multifunctional enzyme
POS	Positive
PPT	Palmitoyl-protein thioesterase
PVDF	Polyvinylidene fluoride
QC	Quality control
QTOF	Quadrupole time-of-flight
RNA	Ribonucleic acid
RT	Retention time
S02	Non-AHPND-causing strain
S	Supernatant
SCP	Sterol carrier protein
SDS-PAGE	Sodium dodecyl sulfate polyacrylamide gel electrophoresis
T3SS2	Type III secretion system 2
TBST	Tris buffered saline tween-20
TCBS	Thiosulfate citrate bile salts sucrose
TCA	Taurocholic acid
TCDCA	Taurochenodeoxycholic acid
TSB	Tryptic soy broth
UHPLC	Ultra-high-performance liquid chromatography
vp	*Vibrio parahaemolyticus*
