# Supplementary material for: Metabolic Alterations in Shrimp Stomach During Acute Hepatopancreatic Necrosis Disease and Effects of Taurocholate on Vibrio parahaemolyticus
Source: Front Microbiol. 2021 Apr 20;12:631468. doi: 10.3389/fmicb.2021.631468 (PMC8093816; doi:10.3389/fmicb.2021.631468)

Supplementary Materials

Metabolic alterations in shrimp stomach during acute hepatopancreatic necrosis disease and effects of taurocholate on *Vibrio parahaemolyticus*

Ramya Kumar [1,2], Teng-Chun Tung [1], Tze Hann Ng [2,3], Che-Chih Chang [1], Yi-Lun Chen[1], Yi-Min Chen [1,2], Shih-Shun Lin [4], Han-Ching Wang [1,2]

[1]Department of Biotechnology and Bioindustry Sciences, National Cheng Kung University, Tainan, Taiwan
[2]International Center for Scientific Development of Shrimp Aquaculture, National Cheng Kung University, Tainan, Taiwan
[3]Institute for Evolution and Biodiversity, University of Münster, Münster, Germany
[4]Institute of Biotechnology, National Taiwan University, Taipei, Taiwan

***Corresponding author: Han-Ching Wang**
E-mail: wanghc@mail.ncku.edu.tw
Phone: +886-6-2757575 ext: 58219
Fax: +886-6-276-6490
Address: Department of Biotechnology and Bioindustry Sciences, College of Bioscience and Biotechnology, National Cheng Kung University, Tainan 701, Taiwan

Table S1. Primers used in this study for real-time PCR

Gene	Primer name*	Primer sequence (5'-3')
*Lv*EF1α	EF1α-qF	ACGTGTCCGTGAAGGATCTGAA
	EF1α-qR	TCCTTGGCAGGGTCGTTCTT
*Lv*Δ-9 desaturase	PVHP193509.6-qF	TCGCCCTGCTCCACTTCTA
	PVHP193509.6-qR	TTCCACTGCACCGAGAACAG
*Lv*ACT-1	PVHP220574.1-qF	GATCGGCGCAGCTAGCA
	PVHP220574.1-qR	CGAAAAAGGCAAGGGACAAC
*Lv*ACT-2	PVHP246814.1-qF	GGCGGCCATTCGAACAC
	PVHP246814.1-qR	GTGCTTAACGAGAAAGTTCCTCATC
*Lv*ACT3	PVHP261419.1-qF	CAACCCTCCCCATTGAACAA
	PVHP261419.1-qR	TCGTCACTGCCAACAAGGAA
*Lv*PPT	PVHP177237.1-qF	TGCCCTGAGTTGCCACAA
	PVHP177237.1-qR	TCAGCACTTTGCGGACATATTC
*Lv*Δ-9 desaturase	PVHP193509.6-qF	TCGCCCTGCTCCACTTCTA
	PVHP193509.6-qR	TTCCACTGCACCGAGAACAG
*Lv*ACOX	LvACOX_F	CCCGGGTGTCCACTGACA
	LvACOX_R	GTAGCGTCCGAGGCACTGA
*Lv*AMACR	LvAMACR_F	CCAACACCCCCTATCAACCTT
	LvAMACR_R	TCCCTGATCGACTGCGTTCT
*Lv*BAAT	LvBAAT_F	CACTCCGAGTTCTTGCCTTCA
	LvBAAT_R	GGTATCCTTCATGGACGAGTACAGA
*Lv*PMFE	LvPMFE_F	AAGTTTGGCCGCATCATCAT
	LvPMFE_R	TCAAGCCAAGCAGACCAAGTT
*Lv*SCP	LvSCP_F	GCGCTTTCTGAAGATGGACCTA
	LvSCP_R	CAATCCACTTGGCTTCTTTGC

* Numbers in the primer name refer to the contig numbers of the corresponding sequences in our in-house transcriptomic database.

Table S2. Quantities of metabolites identified using MS2 database in POS ion mode

Positive ion mode							
**Metabolites**	**Group**	**#1**	**#2**	**#3**	**#4**	**#5**	**#6**
**4-Oxoretinol**	**TSB**	**0.01764**	**0.0190407**	**0.014894**		**0.014451**	**0.011725**
	**S02**	**0.01801**	**0.0111462**	**0.014316**	**0.012556**	**0.013147**	**0.018521**
	**5HP**	**0.063371**	**0.0485453**	**0.064268**	**0.06913**	**0.049841**	**0.065108**
**(4Z,7Z,10Z,13Z,16Z,19Z)-4,7,10,13,1 6,19-Docosahexaenoic acid**	**TSB**	**0.002186**	**0.0021889**	**0.001936**		**0.002021**	**0.002711**
	**S02**	**0.002107**	**0.0034324**	**0.000166**	**0.0025**	**0.003011**	**0.002543**
	**5HP**	**0.018924**	**0.0173506**	**0.023012**	**0.027066**	**0.046288**	**0.011666**
Adenosine 2',3'-cyclic monophosphate	TSB	0.015309	0.0122793	0.011587		0.030722	0.013967
	S02	0.008305	0.0163426	0.0091	0.016929	0.010895	0.016735
	5HP	0.001879	0.0021442	0.002136	0.002078	0.001325	0.001611
Arachidonic Acid (peroxide free)	TSB	0.010885	0.0147914	0.012812		0.010606	0.017524
	S02	0.013605	0.0131972	0.014332	0.016268	0.011974	0.013188
	5HP	0.002164	0.0018421	0.002313	0.003201	0.001648	0.002421
Caffeine	TSB	0.00695	0.0238146	0.007496		0.005767	0.026668
	S02	0.012267	0.0167014	0.013101	0.015215	0.014899	0.015065
	5HP	0.001057	0.0009008	0.001252	0.001884	0.00104	0.00168
Cholesterol	TSB	0.003709	0.0058072	0.007638		0.00617	0.00736
	S02	0.006173	0.0075204	0.004096	0.006395	0.004747	0.005394
	5HP	0.00043	0.0006955	0.00057	0.000454	0.000277	0.000487
D-Alanyl-D-alanine (D-Ala-D-Ala)	TSB	0.042546	0.0387806	0.038171		0.032859	0.038299
	S02	0.026258	0.040403	0.042441	0.036955	0.032865	0.040111
	5HP	0.001925	0.0022843	0.002305	0.002151	0.001961	0.00291
Deoxycytidine	TSB	0.014906	0.0253263	0.02207		0.029694	0.025853
	S02	0.008454	0.0436499	0.016245	0.018533	0.049197	0.041765
	5HP	0.00153	0.0038866	0.003688	0.002459	0.002265	0.003679
Indoleacetic acid	TSB	0.001994	0.0025665	0.002279		0.003229	0.0025
	S02	0.001965	0.0049493	0.002199	0.002196	0.002103	0.003535
	5HP	0.000487	0.0006364	0.000536	0.000478	0.00041	0.000806
L-homoserine	TSB	0.006029	0.0070216	0.006499		0.004623	0.00705
	S02	0.005613	0.0078236	0.005975	0.004365	0.005321	0.005259
	5HP	0.000933	0.0017106	0.001501	0.001723	0.001189	0.001884
Sarcosine	TSB	0.030846	0.0295894	0.022341		0.016762	0.029052
	S02	0.023069	0.0198471	0.030877	0.017645	0.019835	0.019293
	5HP	0.001101	0.0009683	0.000859	0.001297	0.001059	0.001244
Taurochenodeoxycholate	TSB	0.006965	0.0065152	0.012851		0.015365	0.010011
	S02	0.009691	0.0173527	0.008562	0.007833	0.011057	0.016535
	5HP	0.000665	0.0007973	0.000723	0.000669	0.000526	0.000422
Taurocholate	TSB	0.079003	0.1015854	0.182313		0.171579	0.163379
	S02	0.124578	0.2072673	0.070258	0.12289	0.158137	0.125344
	5HP	0.00203	0.0036343	0.002173	0.003231	0.002172	0.003053

Metabolite quantities that were increased in 5HP group are highlighted in red.
Metabolite quantities that were decreased in 5HP group are highlighted in green.

Table S3. Quantities of metabolites identified using MS2 database in NEG ion mode

Negative ion mode							
**Metabolite**	**Group**	**#1**	**#2**	**#3**	**#4**	**#5**	**#6**
Oleic acid	TSB	0.421659	0.162056	0.162744		0.136789	0.530289
	S02	0.172115	0.4235	0.146468	0.25377695	0.662487	0.531849
	5HP	8.030137	11.53936	12.16587	7.38159949	10.18534	10.25268
Palmitic acid	TSB	1.832479	1.134898	1.197628		1.292599	0.944494
	S02	0.883525	0.894693	0.927842	0.99932219	1.293753	0.92892
	5HP	15.93636	18.48048	24.37039	15.7738762	17.63064	20.81887
Arachidic acid	TSB	0.017116	0.014636	0.015844		0.01407	0.008222
	S02	0.012761	0.008157	0.014587	0.00995578	0.016654	0.007708
	5HP	0.0654	0.076548	0.120316	0.07551845	0.098715	0.130824
Erucic acid	TSB	0.007667	0.004572	0.007281		0.005356	0.006596
	S02	0.005201	0.004647	0.003477	0.00670401	0.006267	0.007583
	5HP	0.111603	0.294198	0.332162	0.1111838	0.206145	0.359671
Linoleic acid	TSB	0.184913	0.110904	0.11039		0.213305	0.290926
	S02	0.115352	0.223724	0.246748	0.13746242	0.221463	0.107235
	5HP	7.489796	9.559209	8.522549	6.81988128	8.226778	7.509113
Palmitaldehyde	TSB	0.01099	0.013752	0.011811		0.005096	0.00786
	S02	0.005888	0.008077	0.012764	0.00745604	0.007113	0.007521
	5HP	0.082639	0.066	0.07158	0.06997238	0.106872	0.059235
11beta-Hydroxyprogesterone	TSB	0.003414	0.003136	0.004091		0.003629	0.002893
	S02	0.00451	0.005777	0.002817	0.00503975	0.003322	0.003711
	5HP	0.000593	0.001103	0.000867	0.0005906	0.000598	0.00109
Acetylcarnitine	TSB	0.00697	0.022402	0.01776		0.015673	0.070345
	S02	0.005788	0.041551	0.012192	0.03297698	0.015674	0.050542
	5HP	0.000684	0.00129	0.00163	0.00047394	0.000717	0.000727
Capric acid	TSB	0.004224	0.005272	0.005879		0.006465	0.008638
	S02	0.003578	0.011494	0.005019	0.0048621	0.007605	0.008107
	5HP	0.00041	0.000489	0.000438	0.00039721	0.000362	0.000476
Coniferol	TSB	0.014949	0.018263	0.016607		0.032331	0.018704
	S02	0.014447	0.028781	0.012154	0.02879517	0.018069	0.021406
	5HP	0.003577	0.005634	0.004575	0.00382464	0.000403	0.003731
Cortisone	TSB	0.015302	0.018794	0.017947		0.02482	0.018185
	S02	0.010616	0.035777	0.014289	0.02563632	0.016658	0.037496
	5HP	0.001942	0.004162	0.002578	0.00245232	0.001366	0.001143
Creatine	TSB	0.058038	0.069633	0.072176		0.103609	0.094755
	S02	0.062266	0.129599	0.078963	0.08990557	0.09842	0.098768
	5HP	0.0141	0.017039	0.015916	0.01733226	0.014154	0.018236
Guanosine	TSB	0.010656	0.00903	0.007696		0.023174	0.011002
	S02	0.008236	0.01473	0.010646	0.01654545	0.012494	0.01674
	5HP	0.00165	0.002682	0.001772	0.00173702	0.001685	0.001294
L-Carnitine	TSB	0.00573	0.007655	0.010229		0.007874	0.008287
	S02	0.007583	0.013835	0.015492	0.01216315	0.008421	0.012698
	5HP	0.002433	0.002041	0.002349	0.00208811	0.002281	0.001834
Mevalonic acid	TSB	0.209108	0.298507	0.387391		0.42641	0.399528
	S02	0.406278	0.484493	0.297061	0.39049934	0.343199	0.415283
	5HP	0.004736	0.008631	0.008734	0.012206	0.004674	0.020947
N-Acetyl-D-Glucosamine 6-Phosphate	TSB	0.536391	0.955353	0.562071		0.544428	0.574782
	S02	0.924987	0.580815	0.375586	0.98573673	0.598387	0.640437
	5HP	0.058001	0.059965	0.065946	0.05181385	0.0558	0.066693
Taurocholate	TSB	0.329985	0.428563	0.723467		0.757327	0.835063
	S02	0.538572	0.905482	0.287832	0.547833	0.693764	0.600234
	5HP	0.004587	0.011014	0.006711	0.005258	0.001951	0.008136
Taurochenodeoxycholate	TSB	0.546031	0.570194	1.100001		1.126778	1.865743
	S02	1.260987	1.835989	0.4389245	1.463774	1.153202	1.50787
	5HP	0.004587	0.011260	0.008735	0.007664	0.010768	0.009022

Metabolite quantities that were increased in 5HP group are highlighted in red.
Metabolite quantities that were decreased in 5HP group are highlighted in green**.**

Fig. S1. AHPND diagnosis results for the 5HP-challenged shrimp stomachs collected at (A) 12 hpi and (B) 24 hpi by using IQ2000 AHPND/EMS Toxin 1 Detection and Prevention System. The presence of bands with the sizes of 218 bp and 432 bp implied that all of these shrimp were infected with AHPND.

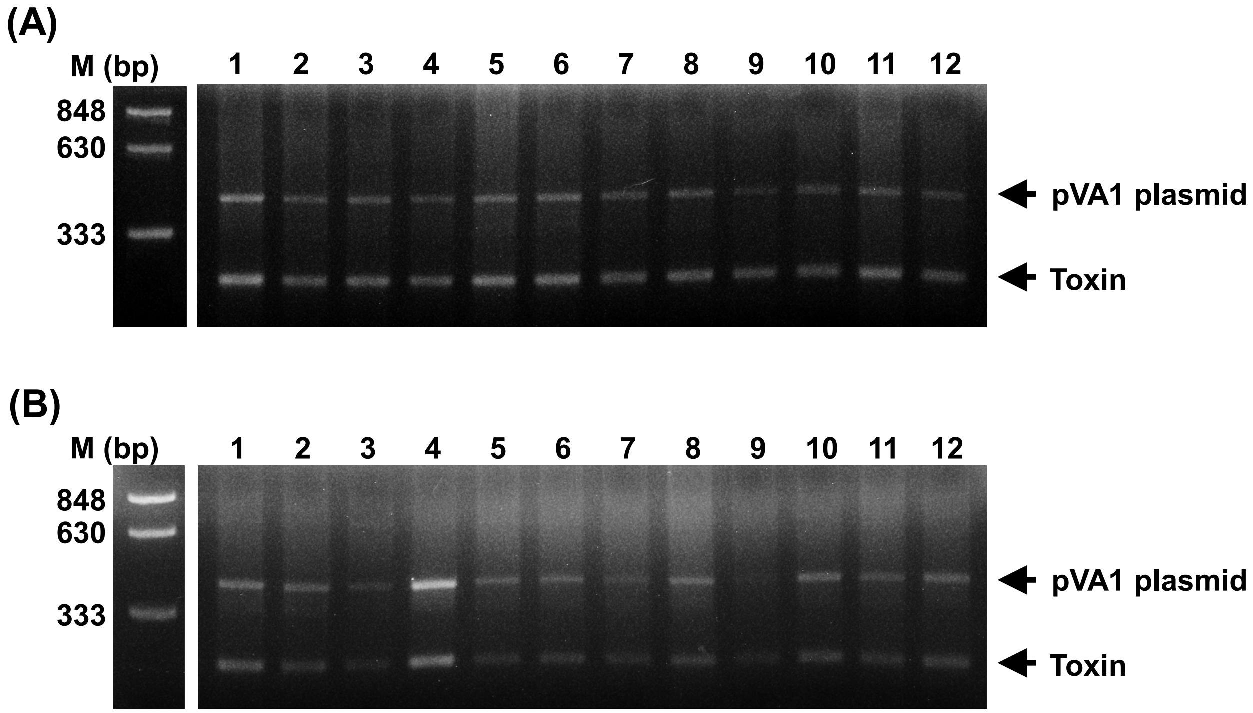

Supplement: Supplementary file 1 [file Data_Sheet_1.pdf]
